# Influenza

**Published:** 1890-01-18

**Authors:** 


### INFLUENZA.

Influenza. How tired everyone is by this time of hearing this word repeated time after time during the past few weeks, and yet what a boon the subject must have proved to Englishmen as a topic of conversation when the weather topic had utterly failed. The *Universal Review* has this month an article on this subject by Dr. R. Sisley, of which we have seen an advance proof. Despite, therefore, our readers' weariness of the subject, we ask them to bear with us while we give a short sketch of this epidemic which has caused such inconvenience during the last few months.

Influenza—and by this we mean the epidemic which has paid a visit in past years to most parts of the world—has a record stretching far back, how far back it is hard to say, but, at any rate, in 1580 mention is made of it. It is highly infectious, and is remarkable for the extreme celerity with which the infection spreads, the great number of people affected, and the, happily, comparatively small number of fatalities consequent upon it. Another distinguishing feature of it is that, once started, it visits country after country until it has been to nearly every nation on this earth. England, during the past century, has been visited by it on three occasions—viz., in 1833-37, 1847, and 1889-90—and on these three occasions the symptoms of the disease were practically the same. These are described by Dr. Sisley to be "shivering, a high temperature, great prostration, acute pain in the back and in the muscles and joints, a burning feeling in the eyes, some increase in the flow of tears, occasionally sneezing, and more rarely a discharge from the nose. . . Of these symptoms the intensity of the prostration and of the pain have in many cases been most striking. . . The pain in the back is often very severe, and is followed by a feeling which is like that felt in a severe bruise. . . Sore throat is a very common occurrence in influenza; the tonsils are often enlarged and red, and there are frequently white patches of secretion on them (*folicular tonsillitis*").

The history of the course of the present epidemic is briefly this: Apparently it originated in Siberia, and in October of last year it appeared in St. Petersburg, where it spread so rapidly that something like two-thirds of the population suffered from it. By the end of November it had reached Berlin, and with lightning swiftness attacked the rest of Europe, pushing westward with all speed. On the 11th of December Vienna capitulated, and within the month England, France, and Spain and Portugal had succumbed, and the epidemic had crossed the Atlantic and put in an appearance in America. One marked difference has manifested itself in England in the mode of the beginning of the outbreak. In former years the first appearance and general attack were almost identical, while in the present epidemic influenza has first shown itself, not as a universal infection, but in a number of distinct centres.

As to the cause of the disease opinions differ, and "doctors fall out" on the subject. That maintained by the bacteriologists seems the most feasible one. Dr. Klein thus explains this theory. He says that the primary cause is a microbe, the epidemic character and infectiousness of the disease being adduced as reasons for believing this. It is further suggested that the microbe "is one which multiplies very rapidly, that it is conveyed and that it enters by the breath (*i.e.*, that it spreads by the air), and lastly, that it finds in most persons a suitable nidus for living and thriving." The rapidity with which it spreads from one country to another is taken as a proof that the microbe is air-borne—that is, that it travels with the wind—and, lastly, reasoning from the numerous cases in which, besides the fever and general illness, no other symptom has occurred, it is suggested that the microbe lives and thrives in the blood of the infected person, and that having done its work it is excreted and removed therefrom. "With this harmonises the fact," says Dr. Klein, "that in many cases symptoms on the part of the respiratory organs occur only after the fever has passed off; further, it harmonises with the infectivity of the breath of an affected person; and lastly, with the observation that the stage after the fever, when the respiratory symptoms have become prominent, is the most infective stage."

Now comes the question of the best precautions we should take to keep us free from the disease. These are, says Sir Joseph Fayrer, K.C.S.I., "careful living and clothing, moderation in all things, avoiding depressing influences, fear, fatigue, chills, and violent alterations of temperature. Every attention to symptoms and immediate rest from work, fatigue, and exposure. Needless to say that impure water, foul air, bad drains, and damp subsoil should be guarded against."

